# Spectroscopic analysis of chia seeds

**DOI:** 10.1038/s41598-021-88545-5

**Published:** 2021-04-29

**Authors:** Monica Mburu, Olivier Paquet-Durand, Bernd Hitzmann, Viktoria Zettel

**Affiliations:** 1 Institute of Food Bioresources Technology, Dedan Kimathi University of Technology, Private Bag, Dedan Kimathi, Nyeri, Kenya; 2 Process Analytics and Cereal Science, Institute of Food Science and Biotechnology, University of Hohenheim, Garbenstr. 23, 70599 Stuttgart, Germany

**Keywords:** Fluorescence spectroscopy, Near-infrared spectroscopy, Raman spectroscopy, Characterization and analytical techniques

## Abstract

Chia seeds are becoming more and more popular in modern diets. In this contribution NIR and 2D-fluorescence spectroscopy were used to determine their nutritional values, mainly fat and protein content. 25 samples of chia seeds were analysed, whereof 9 samples were obtained from different regions in Kenya, 16 samples were purchased in stores in Germany and originated mostly from South America. For the purchased samples the nutritional information of the package was taken in addition to the values obtained for fat and protein, which were determined at the Hohenheim Core Facility. For the first time the NIR and fluorescence spectroscopy were used for the analysis of chia. For the spectral evaluation two different pre-processing methods were tested. Baseline correction with subsequent mean-centring lead to the best results for NIR spectra whereas SNV (standard normal variate transformation) was sufficient for the evaluation of fluorescence spectra. When combining NIR and fluorescence spectra, the fluorescence spectra were also multiplied with a factor to adjust the intensity levels. The best prediction results for the evaluation of the combined spectra were obtained for Kenyan samples with prediction errors below 0.2 g/100 g. For all other samples the absolute prediction error was 0.51 g/100 g for fat and 0.62 g/100 g for protein. It is possible to determine the amount of protein and fat of chia seeds by fluorescence and NIR spectroscopy. The combination of both methods is beneficial for the predictions. Chia seeds from Kenya had similar protein and lipid contents as South American seeds.

## Introduction

Chia, *Salvia hispanica* L., belonging to the mint family, Lamiaceae, is a two metre tall herbaceous plant, native to Southern Mexico and Northern Guatemala^1^. The current research on health-promoting properties of food has developed substantial interest in chia due to its dense nutritional composition, and the fact that chia seeds have been consumed for medicinal purposes for thousands of years^2–4^. Chia seeds are being currently utilized for food fortification in baked products^5^, dairy, soups and sources, fruit juices among others^6^. Chia seeds have been related to different medicinal influences, in particular positive effects on hypertension, cardiovascular diseases, antidiabetic and anti-inflammatory activities^7^. This is because chia seeds are rich in bioactive components with health promoting properties but the composition depends on genetic components as well as the agroecological region of growth^8^. Chia seeds are an excellent source of dietary fat (20–34%), with remarkable levels of polyunsaturated fatty acids (up to 60% α-linolenic and 20% linoleic acids^7^). The seeds are rich in dietary fibre (30–34%), with an insoluble fraction of approximately 85–93%, and a soluble dietary fibre approximately 7–15%^9,10^. Furthermore, chia seeds are a good source of plant protein (18–24%), with 10 exogenous amino acids, of which the greatest contents are leucine, phenylalanine, valine, arginine and lysine^11^, and endogenous amino acids, mainly glutamic and aspartic acids, glycine, serine and alanine^11,12^. Chia seeds do not contain gluten and are therefore suitable for celiac patients^2^. The seeds contain high amounts of B vitamins: niacin (883 mg/100 g), folic acid (49 mg/100 g), thiamine (0.62 mg/100 g) and riboflavin (0.17 mg/100 g)^13^. Kulczyński et al.^11^ found chia seeds to contain vitamin E as tocopherols: α-tocopherol (8 mg/kg of lipids), γ-tocopherol (422 mg/kg of lipids) and δ-tocopherol (15 mg/kg of lipids). Additionally, chia seeds are rich in minerals, with calcium (456 mg/100 g to 631 mg/100 g), phosphorus (860 mg/100 g to 919 mg/100 g), potassium (407 mg/100 g to 726 mg/100 g) and magnesium (335 mg/100 g to 449 mg/100 g)^14^. Studies by various researchers also identified interesting groups of high biological activity phyto-compounds^15–17^. These include polyphenols: chlorogenic, cinnamic, gallic, ferulic, p-coumaric and caffeic acids, kaempferol, quercetin, rutin, epicatechin and apigenin; isoflavones, such as glycitein, daidzein, genistin and genistein in small amounts; and sterols such as campesterol, β-sitosterol, stigmasterol, and avenasterol.

Spectroscopy is widely spread in food characterization and control^18,19^. Infrared spectroscopy was for example applied to determine the total phenolic content of rice^20^. NIR-FT-Raman spectroscopy was used to determine the apparent amylose contents of milled rice with a standard error of prediction for protein of 0.138% and 1.05% for apparent amylose. Protein in flour samples was also predicted based on near infrared reflectance^21^.

Vibrational spectroscopy, infrared and Raman spectroscopy have been introduced to chia in food applications, such as chia oil emulsion gels for the production of sausages^22,23^. Infrared radiation is divided into far-, medium- and near infrared. It is located between the wavelength regions of visible light as the lower and microwaves as the upper limit. The measuring principle is that certain molecules are excited into a higher vibrational state, by absorbing electromagnetic radiation. The absorbed energy is then converted in different vibrations of the C–H, O–H, S–H, or N–H groups, which are found throughout all foods. For this reason, NIR spectroscopy is a prominent candidate for the investigation of food properties and chosen for this investigation beside fluorescence spectroscopy.

Fluorescence spectroscopy is well known for its high substance-specific sensitivity and therefore well established in biological sciences and food research^24^. The measuring principle is based on excitation of molecules from ground states to higher electronic and vibrational states by absorbing a photon. As consequence fluorescence light can be emitted when the molecule goes back to the electronic ground state. The emitted fluorescence light has a longer wavelength than the excitation light, because the molecule in the excited electronic state relaxes faster to the vibrational ground state than to the electronic ground state. Taking this into account the sample can be analysed^25^. Different fluorophores present in food systems can therefore be measured by fluorescence spectroscopy, such as proteins, vitamins, coenzymes and chlorophyll.

Spectroscopy is a fast and easy applicable measurement method. The time-consuming part is the calibration procedure to obtain reliable chemometric models for the prediction for the required variables. Usually different pre-processing methods are used to smooth the data, reduce noise and correct the baseline. Well established methods here are: the Savitzky–Golay–Filter^26^ and the multiplicative scatter correction (MSC)^27^ as well as the standard normal variate transformation (SNV)^28^. Partial least squares regression (PLSR), principal component regression (PCR), or artificial neuronal networks (ANN) are used in most cases to correlate the spectra with calibration data^29^.

In the present work, different spectroscopic methods were used to identify major classes of organic compounds in chia seeds followed by chemometric evaluation of spectra taken from different chia seeds. The chia seeds were obtained from diverse agro ecological zones of Kenya, sampled directly from the farms, and compared with chia seeds sold in the German markets mostly originated from South America. For the first time, the nutritional composition was evaluated by fluorescence and NIR spectroscopy in parallel, so that a fast determination is possible. The study reveals essential nutritional and chemical composition that strengthens utilization of chia seeds for human health benefits and as an important ingredient in functional food.

## Materials and methods

For the study, 25 samples of chia seeds were spectroscopically examined. These include 9 samples from Kenya (named A to I) cultivated from different sites. Chia seeds were collected from different regions of Kenya, in accordance with the relevant institutional research policy, DeKUT RESEARCH POLICY, August 2016, and the national guidelines LEGAL NOTICE No. 106, THE SCIENCE, TECHNOLOGY AND INNOVATION ACT, 2013 (No.28 of 2013). The rest was obtained from different local and online markets in Germany originally from Mexico, Bolivia, Paraguay and Argentina (named J to Y, Table 1). Nutrition information according to the product packing, vendor or the distributor is presented in Supplementary Material 1.

**Table 1:** Information about growing region and determined values for fat and protein of African (A-I) and purchased (J-Z) chia seeds.

label	Origin	Fat (g/(00 g)	Protein (g/100 g)
A	Mwea South Ngariama	24.7	31.5
B	Laikipia Makutano	23.4	33.0
C	Thika	19.7	35.8
D	Embu	24.4	31.5
E	Nyeri Thunguma	22.7	32.6
F	Nyeri Matunda	20.4	34.0
G	Kitale Matunda	18.4	35.0
H	Kivaa	24.4	31.8
I	Kirinyaga	20.6	34.9
J	Bolivia	20.7	34.5
K	ParaquayParaguay	21.1	33.4
L	Mexico	25.3	30.1
M	unknown	21.7	32.6
N	ParaquayParaguay	24.9	31.7
O	Bolivia	21.3	33.1
P	Bolivia, ParaquayParaguay	20.7	33.5
Q	Argentinia	20.0	33.6
R	ParaquayParaguay	18.7	32.4
S	Bolivia	20.2	33.4
T	unknown	24.9	31.7
U	ParaquayParaguay	21.8	32.7
V	Boliviaen	23.2	32.5
W	ParaquayParaguay	20.6	35.3
X	ParaquayParaguay	19.7	33.2
Y	unknown	22	32.8

Single determinations performed by the Analytical Chemistry module of the Core Facility Hohenheim.

For samples A to I, from Kenya, this information was not available, the raw fat and protein contents were therefore for all samples determined by the Analytical Chemistry module of the Core Facility Hohenheim. For the Kenyan samples the fatty acid profiles were determined as well and are presented in Supplementary Material 2. The samples for spectroscopic evaluation were ground with a centrifugal mill (ZM 100, Retsch Technology GmbH, Düsseldorf, Germany) at 6000 rpm. The seeds were frozen for at least 24 h before grinding to avoid changes due to high temperatures.

2D-fluorescence spectra were obtained with the BioView sensor (Delta Light & Optics, Hørsholm, Denmark) equipped with a standard port containing a quartz glass window and a xenon lamp. Spectra were obtained in a range between 270 and 550 nm of excitation and 310 nm and 590 nm emission wavelength with 20 nm distance steps. The resulting spectra contained in total measured intensities of 120 wavelength combinations. A fivefold measurement was performed for each sample. The vial was briefly mixed by shaking after each spectrum recorded.

NIR spectroscopy measurements were performed in the Multi-Purpose NIR Analyzer (Bruker Optik GmbH, Ettlingen, Germany), varying wavelengths from 800 to 2800 nm (wavenumbers from 3599 to 12,489 cm^−1^). The flour samples are filled into suitable vials and placed on the reflection position of the NIR spectroscope. A fivefold measurement is performed for each sample. The vial is briefly mixed by shaking after each spectrum recorded.

The evaluation of the spectra was performed with Matlab R2019a (version 9.6). The evaluation was performed for all purchased samples (dataset DS 1), all Kenyan samples (dataset DS 2) and all samples together (dataset DS 3).

The NIR and fluorescence spectra were evaluated individually and also together as a combination dataset. The spectra were pre-processed with different methods to extract the desired information. For pre-processing variant 1 (PP1) standard normal variate transformation (SNV) was performed. For pre-processing variant 2 (PP2) a baseline correction was performed by removing the low frequency parts of the spectra. This was done by smoothening the first derivate of the spectra with a moving average filter (window width: number of points in spectrum divided by 20), then integrating the smoothened first derivate and subsequently subtracting it from the original spectrum. This was applied prior to SNV to NIR spectra. For pre-processing variant 3 (PP3) the fluorescence spectra were additionally multiplied with a factor of 0.25 for scaling.

Where no measurement values for a particular variable were available, the samples were ignored/left out before applying the Principal Component Analysis (PCA) and Partial Least Squares Regression (PLSR). A PCA with 10 principal components was performed. The offline values were then correlated with each of these first 10 principal components subsequently to check whether there are correlations in the datasets to the target values (fat and protein content). The datasets were used for PCA and PLSR evaluations. 1 up to 32 principal components were tested for the PLSR model. For all datasets, single and combined spectra, a cross-validations (CV)^30^ were carried out and the coefficient of determination R^2^ and the root mean square error of prediction RMSEP (absolute error) were calculated. Furthermore, the RMSEP was calculated with respect to the range of the sample values and is named RMSEP_range_ (percentage error).

## Results and discussion

**Figure 1:**
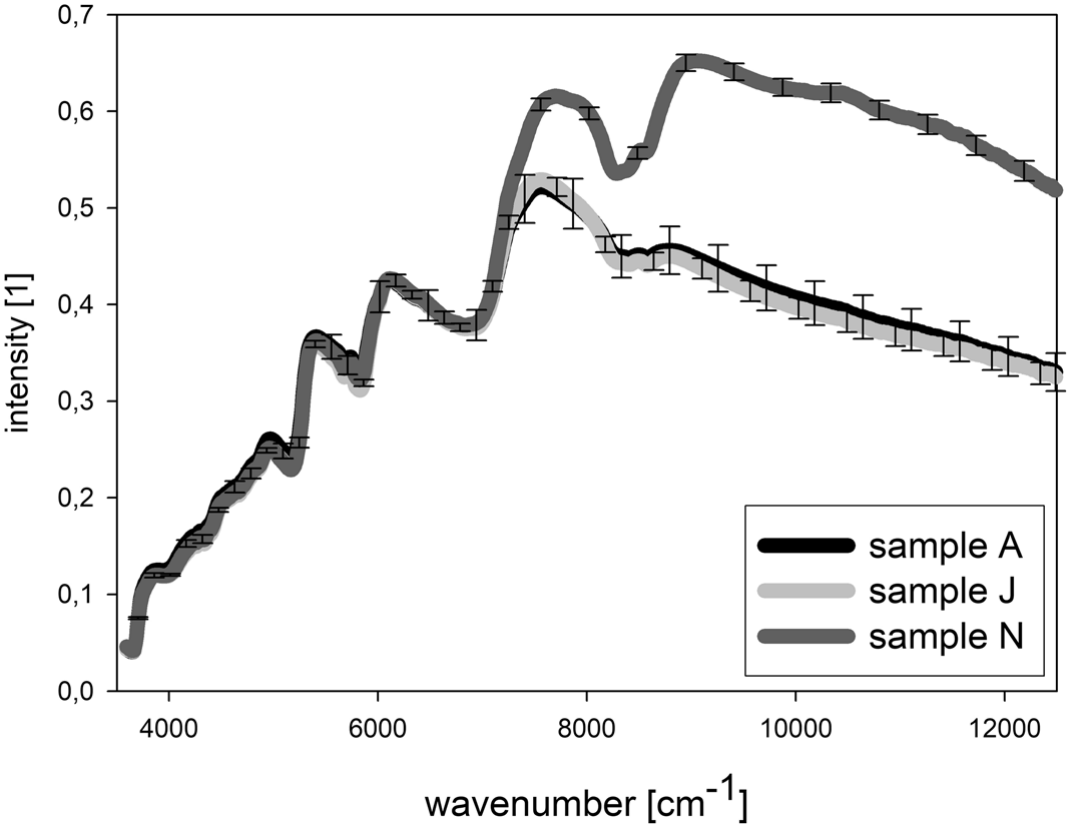
Representative NIR spectra (mean of 5) of three samples before evaluation.

**Figure 2:**
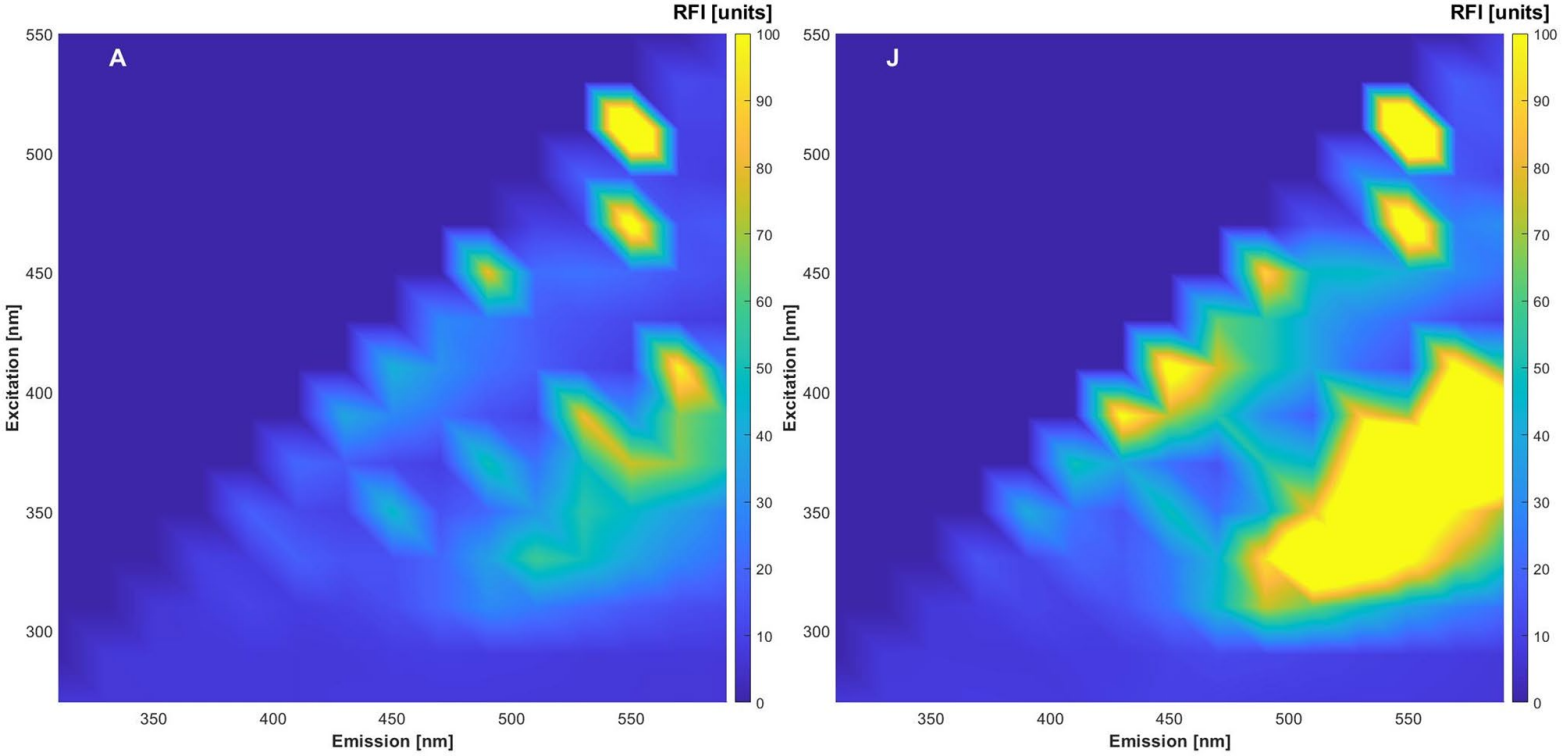
Representative fluorescence spectra (mean of 5) of two samples before evaluation.

**Figure 3:**
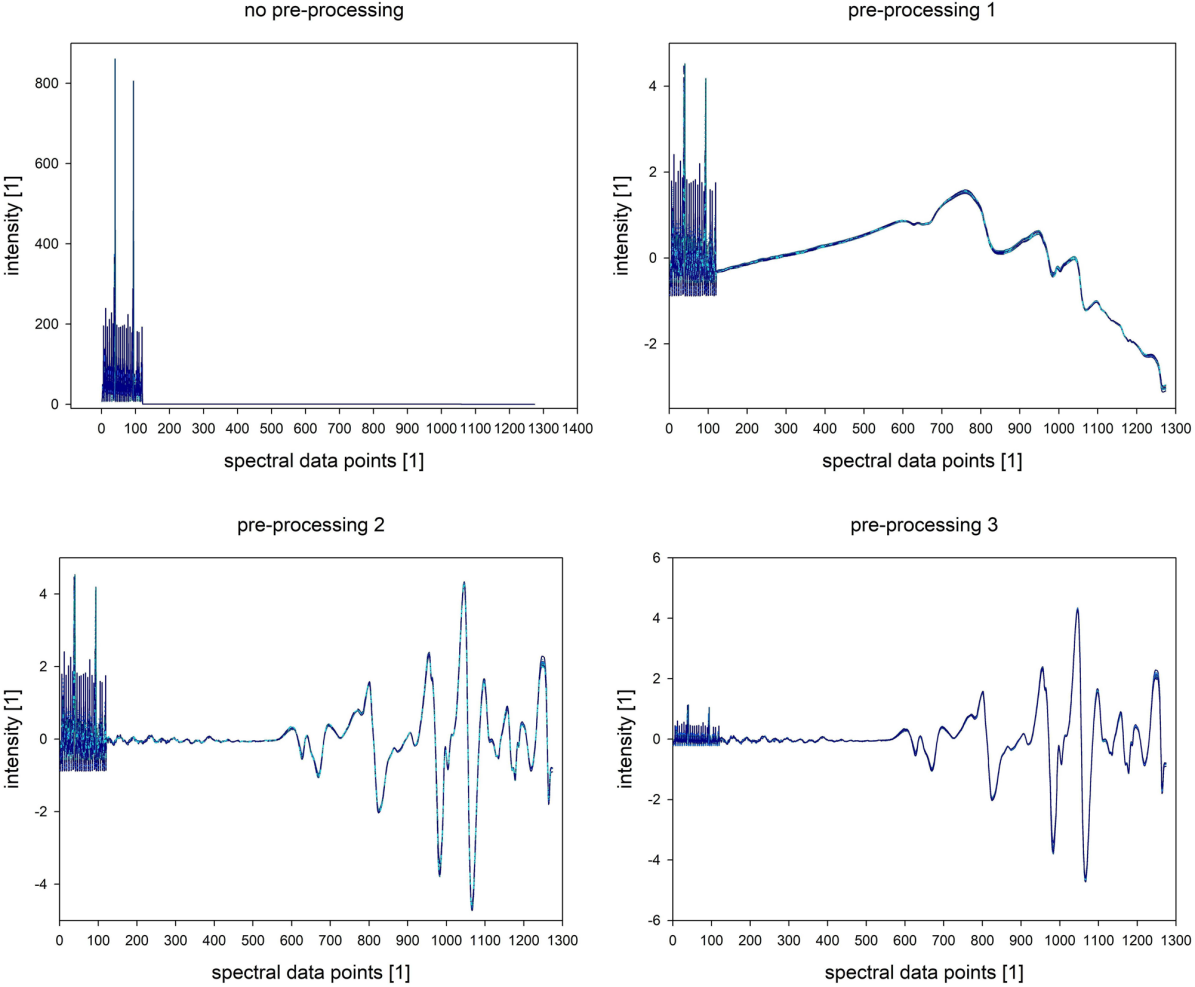
Not and pre-processed spectra of fluorescence (point 1–120) and NIR (121 up) measurements.

Chia seeds from Kenya have similar contents of fat and protein (compare Table 1; Supplementary Material 1) as the South American seeds. Table 1 shows single determinations of protein and fat contents, they range between 18.4–24.7% for fat and 31.5–35.8% for protein for the Kenyan samples which is within the range of the determined values for the ones of Middle and South America. The fatty acid composition is also in the range of the sample from Bolivia, which additional was evaluated as reference (compare Supplementary Material 2). It was expected that the spectra will show similar results. Representative NIR and fluorescence spectra of chia seeds are presented in Figs. 1 and 2, respectively. Sample N is the only sample with white surface of the seeds. There are already differences visible, but they might be due to the inhomogeneous surfaces for both measurement methods. Three different variants of pre-processing were tested. A simple SNV transformation was performed first, so that the spectral data are not too much modified. The results were not satisfying, so that two other pre-processing methods were tested. Raw and pre-processed (all variants) combined fluorescence and NIR spectra are presented in Fig. 3.

The best correlation results of the principal components of the fluorescence or NIR spectra respectively with the measured data are presented in Table 2. Using only fluorescence spectra, coefficients of determination of less than 0.5 were obtained. For evaluated fluorescence spectra with data set DS3 the best results were obtained with no pre-processing with R^2^ = 0.27 for fat and R^2^ = 0.34 for protein, for data set DS1 and DS2 the results were worse. So, no obvious correlation between fat or protein content and the fluorescence data could be found.

**Table 2:** Two best correlation results of the PCA for no and pre-processing methods for the single and combined spectra as well as all datasets (DS).

	noPP		PP1		PP2		PP3	
	Fat	Protein	Fat	Protein	Fat	Protein	Fat	Protein
**Kenyan samples (DS1)**								
Fluorescence								
PC1-2	0.02	0.06	0.26	0.19	0.26	0.19		
PC1-7	0.03	0.00	0.28	0.27	0.28	0.27		
NIR								
PC1-1	0.14	0.33	0.16	0.15	0.72	0.60		
PC1-2	0.14	0.09	0.68	0.59	0.15	0.15		
Combined spectra								
PC1-1	0.01	0.04	0.09	0.04	0.12	0.06	0.71	0.55
PC1-7	0.03	0.00	0.31	0.27	0.07	0.05	0.08	0.10
**Purchased samples (DS2)**								
Fluorescence								
PC1-3	0.15	0.00	0.15	0.13	0.15	0.13		
PC1-8	0.25	0.18	0.43	0.35	0.43	0.35		
NIR								
PC1-2	0.14	0.09	0.04	0.00	0.51	0.12		
PC1-5	0.23	0.02	0.27	0.18	0.15	0.42		
Combined spectra								
PC1-1	0.01	0.04	0.13	0.31	0.08	0.06	0.08	0.19
PC1-2	0.02	0.06	0.02	0.00	0.02	0.03	0.50	0.13
**All samples (DS3)**								
Fluorescence								
PC1-9	0.13	0.34	0.07	0.01	0.07	0.01		
PC1-10	0.27	0.11	0.18	0.10	0.18	0.10		
NIR								
PC1-2	0.17	0.14	0.01	0.03	0.64	0.32		
PC1-3	0.03	0.02	0.53	0.32	0.02	0.23		
Combined spectra								
PC1-2	0.00	0.01	0.06	0.16	0.02	0.01	0.64	0.32
PC1-6	0.00	0.00	0.08	0.18	0.48	0.44	0.01	0.00

R^2^ is the coefficient of determination, RMSEP is the absolute root mean squared error of prediction, RMSEP_range_ is the percentage error. PP indicates pre-processing, PP1: SNV, PP2: SNV and baseline correction, PP3: SNV, baseline correction and reducing factor for fluorescence spectra. PP is for pre-processing.

As NIR spectroscopy is well established for protein and fat determination in food, the coefficients of determination were better here. For NIR PP2 lead to best results for DS1 with R^2^ = 0.72 for fat and R^2^ = 0.6 for protein. For DS2 and DS3 there were still correlations possible with PP2. Applying a baseline correction to the NIR spectra lead therefore to a small improvement of the results compared to PP1 where only a SNV was performed. As expected, the correlations for combined spectra are worse than the solely NIR, but better as the solely fluorescence spectra correlations. The PCA proofed that correlations can be found. The best results of the cross-validated PLSR models are presented in Table 3.

**Table 3:** Results of the PLSR model prediction of fat and protein of chia seeds with single and combined spectra of fluorescence and NIR with no and the three pre-processing variations and all three datasets (DS).

	Fluorescence		NIR			Combined spectra			
	noPP	PP1	noPP	PP1	PP2	noPP	PP1	PP2	PP3
**Kenyan samples (DS1)DS1**									
Fat									
R^2^	0.97	0.99	0.97	0.97	0.94	0.97	0.99	0.99	0.94
RMSEP (g)	0.27	0.12	0.27	0.25	0.38	0.27	0.13	0.15	0.38
RMSEP_range_ (%)	6.19	2.74	6.18	5.90	8.87	6.19	2.99	3.59	8.87
Protein									
R^2^	0.97	1.00	0.94	0.93	0.93	0.97	0.99	0.99	0.93
RMSEP (g)	0.37	0.15	0.55	0.59	0.59	0.37	0.19	0.25	0.59
RMSEP_range_ (%)	5.91	2.39	8.67	9.31	9.27	5.92	2.97	4.02	9.27
**Purchased samples (DS2)DS2**									
Fat									
R^2^	0.78	0.83	0.81	0.76	0.76	0.79	0.92	0.92	0.87
RMSEP (g)	0.56	0.48	0.50	0.57	0.56	0.55	0.32	0.33	0.42
RMSEP_range_ (%)	10.81	9.15	9.61	10.87	10.76	10.51	6.13	6.27	8.12
Protein									
R^2^	0.78	0.83	0.81	0.76	0.76	0.79	0.92	0.92	0.87
RMSEP (g)	0.56	0.48	0.50	0.57	0.56	0.55	0.32	0.33	0.42
RMSEP_range_ (%)	10.81	9.15	9.61	10.87	10.76	10.51	6.13	6.27	8.12
**All samples (DS3)DS3**									
Fat									
R^2^	0.52	0.61	0.82	0.82	0.82	0.53	0.82	0.83	0.85
RMSEP (g)	0.95	0.87	0.56	0.57	0.56	0.95	0.58	0.54	0.51
RMSEP_range_ (%)	16.67	15.35	9.82	9.96	9.78	16.63	10.19	9.51	8.98
Protein									
R^2^	0.61	0.72	0.87	0.87	0.88	0.62	0.91	0.89	0.90
RMSEP (g)	1.30	1.15	0.73	0.73	0.70	1.29	0.62	0.67	0.63
RMSEP_range_ (%)	18.75	16.60	10.54	10.58	10.18	18.65	9.00	9.71	9.11

R^2^ is the coefficient of determination, RMSEP is the absolute root mean squared error of prediction, RMSEP_range_ is the percentage error. PP indicates pre-processing, PP1: SNV, PP2: SNV and baseline correction, PP3: SNV, baseline correction and reducing factor for fluorescence spectra. PP is for pre-processing.

**Figure 4:**
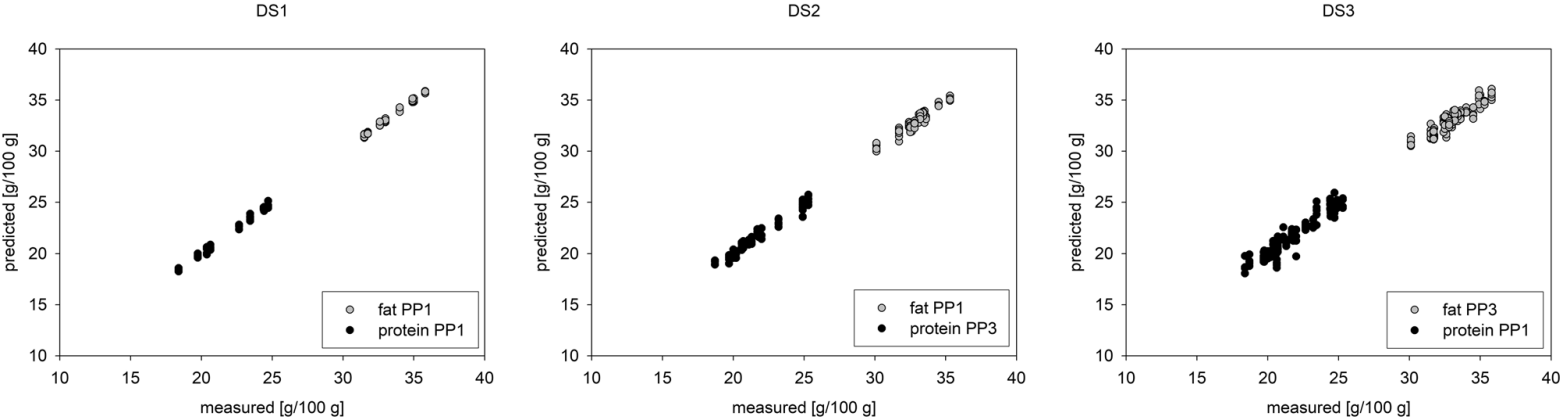
Prediction of fat and protein contents from not and pre-processed spectra of fluorescence (point 1–120) and NIR (121 up) measurements.

The evaluation of only fluorescence and combined spectra obtained best results for the PLSR prediction with PP1 for fat and protein for DS1. For DS2 PP1 was found to be the best pre-processing method for solely fluorescence and NIR evaluations too, but the results were improved by combining fluorescence and NIR spectra. For fat PP1 remained to be the best (R^2^ = 0.92), but for protein PP3 was found to be best (R^2^ = 0.97). The combined evaluation improved the results for DS3 too compared to the poor single evaluations of fluorescence spectra which achieved only R^2^ of 0.61 for fat and 0.72 for protein with PP1 and R^2^ for fat (R^2^ = 0.82) and protein (R^2^ = 0.88) by NIR by PP2. The combination resulted in R^2^ of 0.85 for fat (PP3) and 0.91 for protein (PP1). The best PLSR prediction results for the determined fat and protein contents are depicted in Fig. 4. Taking the given nutritional values of the manufacturers/vendors of the chia seeds into account the best prediction results were obtained for saturated fatty acids and dietary fibre (R^2^ = 0.97) for combined spectra as presented in Table 4. Individual spectra evaluation for fluorescence lead to good results R^2^ > 0.9 for energy (kcal), fat and saturated fatty acids whereas for NIR lead to R^2^ > 0.8 for dietary fibre and protein. The nutritional values given by the distributors are average values, which are not determined and changed for every charge, so it is comprehensible, that the prediction results are worse compared to the values determined for the other samples.

**Table 4:** Best PLSR prediction results for purchased samples (I-Y, DS1) with the given nutritional values by the manufacturers.

	Energy	fat	Saturated fatty acids	Carbohydrates	Dietary fibre	Protein
**noPP combined spectra**						
R^2^	0.67	**0.92**	**0.97**	0.79	0.82	0.88
RMSEP	169 kJ	0.49 g	0.26 g	4.83 g	1.63 g	0.44 g
RMSEP_range_ (%)	16	8.08	3.81	12.87	15.22	8.97
**PP1 combined spectra**						
R^2^	0.74	0.91	0.94	**0.89**	**0.97**	**0.93**
RMSEP	139 kJ	0.52 g	0.38 g	3.52 g	0.65 g	0.34 g
RMSEP_range_ (%)	13	8.51	5.49	9.38	6.07	6.87
**PP2 combined spectra**						
R^2^	0.80	0.89	0.86	0.78	0.95	0.85
RMSEP	116 kJ	0.56 g	0.59 g	4.82 g	0.80 g	0.48 g
RMSEP_range_ (%)	11	9.11	8.59	12.86	7.52	9.79
**PP3 combined spectra**						
R^2^	**0.81**	0.87	0.69	0.70	0.93	0.73
RMSEP	115 kJ	0.59 g	0.87 g	5.69 g	0.96 g	0.63 g
RMSEP_range_ (%)	11	9.69	12.64	15.16	8.98	12.87
**noPP fluorescence**						
R^2^	**0.66**	**0.92**	**0.97**	**0.79**	**0.81**	**0.88**
RMSEP	170 kJ	0.49 g	0.26 g	4.91 g	1.65 g	0.44 g
RMSEP_range_ (%)	16	8.10	3.80	13.09	15.44	9.01
**PP1 fluorescence**						
R^2^	0.59	0.83	0.92	0.70	0.70	0.86
RMSEP	179 kJ	0.68 g	0.46 g	6.32 g	2.42 g	0.48 g
RMSEP_range_ (%)	17	11.21	6.61	16.85	22.63	9.87
**PP2 fluorescence**						
R^2^	0.59	0.83	0.92	0.70	0.70	0.86
RMSEP	179 kJ	0.68 g	0.46 g	6.32 g	2.42 g	0.48 g
RMSEP_range_ (%)	17	11.21	6.61	16.85	22.63	9.87
**noPP NIR**						
R^2^	0.55	0.53	**0.69**	**0.62**	0.82	**0.82**
RMSEP	177 kJ	1.15 g	0.87 g	6.36 g	1.58 g	0.52 g
RMSEP_range_ (%)	17	18.81	12.67	16.95	14.74	10.52
**PP1 NIR**						
R^2^	**0.59**	**0.55**	0.68	0.56	**0.85**	0.78
RMSEP	168 kJ	1.12 g	0.88 g	6.89 g	1.41 g	0.57 g
RMSEP_range_ (%)	16	18.32	12.82	18.37	13.22	11.58
**PP2 NIR**						
R^2^	0.37	0.20	0.39	0.40	0.79	0.46
RMSEP	211 kJ	1.57 g	1.26 g	8.06 g	1.69 g	0.90 g
RMSEP_range_ (%)	20	25.69	18.25	21.51	15.75	18.45

R^2^ is the coefficient of determination, RMSEP is the absolute root mean squared error of prediction, RMSEP_range_ is the percentage error. PP is indicatesfor pre-processing, PP1: SNV, PP2: SNV and baseline correction, PP3: SNV, baseline correction and reducing factor for fluorescence spectra. Best coefficients of determination of the evaluation per data sets are presented in bold.

It was proven that the prediction of nutritional values for Chia seeds is possible by fluorescence and NIR spectroscopy, and the combination of both methods improved the results. However, increasing the range of nutrient diversity or selecting samples with higher variation could improve the prediction results.

## Conclusion

The presented results show that combined evaluation of NIR and fluorescence spectra is suitable to predict nutritional values of chia seeds. The best prediction results were, as expected, obtained for fat and protein with combined spectra. The RMSEP for fat was 0.51 g/100 g and for protein was 0.62 g/100 g (8.98% and 9% respectively calculated with respect to the sample range) for all samples. For Kenyan samples only, the best prediction errors were 0.13 g/100 g for fat and 0.19 g/100 g for protein (2.99% and 2.97% respectively calculated with respect to the sample range). For only purchased samples the errors were 0.32 g/100 g for fat and protein (6.13% calculated with respect to the sample range). For the nutritional values given by the distributors of the purchased chia seeds, the prediction results for fat, thereof saturated fatty acids and protein were the best with prediction errors below 0.7 g/100 g (calculated with respect to the sample range below 10%), which is found to be good compared to the other values. Further studies are necessary to improve the prediction qualities. It is expected that if the range of nutritional and chemical composition of the samples would be increased, the prediction error will be reduced. Furthermore, alternative pre-processing and evaluation methods might lead to better results too. However, due to the fast determination of the nutritional and chemical composition of the samples using the spectroscopic method, it is a promising alternative to the current standard methods.

## Supplementary Information

Supplementary Information 1.

Supplementary Information 2.
