# Supplementary material for: Spectroscopic analysis of chia seeds
Source: Sci Rep. 2021 Apr 29;11:9253. doi: 10.1038/s41598-021-88545-5 (PMC8085002; doi:10.1038/s41598-021-88545-5)
Supplement: Supplementary file 1 — Supplementary Information 1. [file 41598_2021_88545_MOESM1_ESM.docx]

# Spectroscopic analysis of chia seeds

Monica Mburu*, Olivier Paquet-Durand**, Bernd Hitzmann** & Viktoria Zettel**

*Institute of Food Bioresources Technology, Dedan Kimathi University of Technology, Private Bag, Dedan Kimathi, Nyeri, Kenya

**Process Analytics and Cereal Science, Institute of Food Science and Biotechnology, University of Hohenheim, Garbenstr. 23, Stuttgart 70599, Germany

Table 1: Nutrition information (per 100 g) according to the distributor/package for the purchased samples J - K, mainly originated from Middle and South America.

| label | origin | energy [kJ] | fat [g] | sat. fatty acids [g] | Carbo-hydrates [g] | dietary fibre [g] | protein [g] | Vendor |
| --- | --- | --- | --- | --- | --- | --- | --- | --- |
| J | Bolivia | 1968 | 33.4 | 3.9 | 7.9 | 28 | 22 | Vita+Naturprodukte GmbH, Langkampfen, Austria |
| K | Paraguay | 1869 | 32 | 3.5 | 5.2 | 30 | 21 | CDF Sports & Health Solutions GmbH |
| L | Mexico | 1911 | 34 | 3.3 | 0.9 | 38 | 20 | borchers fine food GmbH & Co. KG, Oyten, Germany |
| M | unknown | 1907 | 34.1 | 3.7 | 0.5 | 36.5 | 20.5 | Alnatura GmbH, Bickenbach, Germany |
| N | Paraguay | 1869 | 32 | 4.1 | 5.2 | 30 | 21 | MySuperfoods Ltd, Canterbury, United Kingdom |
| O | Bolivia | 1770 | 28 | 3 | 7 | 31 | 21 | L-Carb-Shop UG, Offenbach, Germany |
| P | Bolivia, Paraguay | 1832 | 31.4 | 3.8 | 4.9 | 33.7 | 21.2 | Activ Organic Limided, Reading, United Kingdom |
| Q | Argentina | 1832 | 31.4 | 3.8 | 38 | 33.7 | 21.2 | Ingo Steyer KG, Hemmoor, Germany |
| R | Paraguay | 1911 | 34 | 3.3 | 0.9 | 38 | 20 | Sevenhills Wholefoods, Wakefield, United Kingdom |
| S | Bolivia | 2787 | 30.4 | 3.2 | 2.3 | 33.5 | 22.5 | Amazon EU S.a.r.l, Luxembourg, Luxembourg |
| T | unknown | 2442 | 32.5 | 9.9 | 29.8 | 38.7 | 24.9 | Wohltuer GmbH, coburg, Germany |
| U | Paraguay | 1832 | 31.4 | 3.8 | 4.9 |  | 21.2 | Alnatura GmbH, Bickenbach, Germany |
| V | Bolivia | 1953 | 34 | 4.1 | 5.3 | 29 | 22 | Ingo Steyer KG, Hemmoor, Germany |
| W | Paraguay | 1723 | 33.1 | 3.5 | 6.4 | 29 | 22.1 | VM Trading GmbH, Wörnitz, Germany |
| X | Paraguay | 1911 | 34 | 3.3 | 0.9 | 38 | 20 | Sportbedarf Christian Jeske, Hünxe, Germany |
| Y | unknown | 1911 | 34 | 3.3 | 0.9 | 38 | 20 | Vita+Naturprodukte GmbH, Langkampfen, Austria |
