# Supplementary material for: Spectroscopic analysis of chia seeds
Source: Sci Rep. 2021 Apr 29;11:9253. doi: 10.1038/s41598-021-88545-5 (PMC8085002; doi:10.1038/s41598-021-88545-5)
Supplement: Supplementary file 2 — Supplementary Information 2. [file 41598_2021_88545_MOESM2_ESM.docx]

# Spectroscopic analysis of chia seeds

Monica Mburu*, Olivier Paquet-Durand**, Bernd Hitzmann** & Viktoria Zettel**

*Institute of Food Bioresources Technology, Dedan Kimathi University of Technology, Private Bag, Dedan Kimathi, Nyeri, Kenya

**Process Analytics and Cereal Science, Institute of Food Science and Biotechnology, University of Hohenheim, Garbenstr. 23, Stuttgart 70599, Germany

Table 2: Fatty acid composition of Kenyan chia seed samples (A-I) and one purchased sample from Bolivia (J).Fatty acid pattern (normalisation method - the value determined is expressed as % fatty acid in relation to total fatty acids). Single determination.

|  |  |  |  | A | B | C | D | E | F | G | H | I | J |
| --- | --- | --- | --- | --- | --- | --- | --- | --- | --- | --- | --- | --- | --- |
| C4:0 | **Butanoic acid** | Butyric acid | % | 0,024 | 0,025 | 0,034 | 0,023 | 0,022 | 0,042 | 0,02 | 0,029 | 0,016 | 0,025 |
| C8:0 | **Octanoic acid** | Caprylic acid | % | 0,016 | 0,016 | 0,013 | 0,013 | 0,018 | 0,013 | 0,015 | 0,02 | 0,012 | 0,017 |
| C14:0 | **Tetradecanoic acid** | Myristic acid | % | 0,061 | 0,048 | 0,041 | 0,058 | 0,05 | 0,04 | 0,039 | 0,06 | 0,045 | 0,055 |
| C15:0 | **Pentadecanoic acid** | | % | 0,03 | 0,026 | 0,021 | 0,029 | 0,025 | 0,021 | 0,021 | 0,029 | 0,021 | 0,026 |
| C16:0 | **Hexadecanoic acid** | Palmitic acid | % | 8,063 | 7,301 | 7,251 | 8,006 | 6,879 | 7,309 | 6,851 | 8,126 | 7,263 | 8,012 |
| C16:1 | **cis-9-hexadecaenoic acid** | Palmitoleic acid | % | 0,064 | 0,058 | 0,065 | 0,065 | 0,058 | 0,066 | 0,059 | 0,065 | 0,064 | 0,07 |
| C17:0 | **Heptadecanoic acid** | Margaric acid | % | 0,07 | 0,057 | 0,049 | 0,07 | 0,055 | 0,049 | 0,046 | 0,066 | 0,052 | 0,059 |
| C18:0 | **Octadecanoic acid** | Stearic acid | % | 4,416 | 3,76 | 3,056 | 4,42 | 4,174 | 2,945 | 3,328 | 4,46 | 3,403 | 3,683 |
| C18:1n9c | **cis-9- Octadecaenoic acid** | Oleic acid | % | 9,736 | 7,264 | 5,485 | 9,778 | 9,612 | 5,07 | 6,034 | 9,829 | 5,844 | 7,076 |
| C18:2n6c | **cis.cis-9.12- Octadecadiensäure** | **Linoleic acid** | **%** | **21,708** | **18,373** | **18,507** | **21,872** | **22,288** | **18,398** | **18,599** | **21,901** | **17,467** | **20,261** |
| C18:3n3 | **all cis-9.12.15- Octadecatrienoic acid** | **α-linolenic acid** | **%** | **54,812** | **62,165** | **64,685** | **54,747** | **55,885** | **65,236** | **64,155** | **54,518** | **65,041** | **59,922** |
| C18:3n6 | **all cis-6-9-12- Octadecatrienoic acid** | γ-linolenic acid | % | 0,085 | 0,081 | 0,058 | 0,083 | 0,099 | 0,055 | 0,07 | 0,085 | 0,053 | 0,065 |
| C20:0 | **Eicosanoic acid** | Arachidic acid | % | 0,342 | 0,287 | 0,256 | 0,343 | 0,329 | 0,255 | 0,281 | 0,344 | 0,283 | 0,289 |
| C20:1 | **cis-11- Eicosanoic acid** | Gondoic acid | % | 0,235 | 0,256 | 0,244 | 0,228 | 0,253 | 0,291 | 0,222 | 0,262 | 0,194 | 0,23 |
| C20:2 | **cis.cis-11.14- Eicosadienoic acid** | | % | 0,034 | 0,043 | 0,03 | 0,03 | 0,032 | 0,027 | 0,03 | 0,026 | 0,031 | 0,025 |
| C20:3n6 | **all cis-8.11.14- Eicosatrienoic acid** | Homo-γ-linolenic acid | % | 0,029 | 0,04 | 0,042 | 0,028 | 0,029 | 0,031 | 0,035 | 0,026 | 0,038 | 0,038 |
| C22:0 | **Docosanoic acid** | Behenic acid | % | 0,111 | 0,094 | 0,084 | 0,081 | 0,076 | 0,069 | 0,085 | 0,061 | 0,08 | 0,059 |
| C24:0 | **Tetracosanoic acid** | Lignoceric acid | % | 0,162 | 0,106 | 0,082 | 0,126 | 0,116 | 0,083 | 0,109 | 0,095 | 0,093 | 0,088 |
